# Supplementary material for: Hybrid Models and Biological Model Reduction with PyDSTool
Source: PLoS Comput Biol. 2012 Aug 9;8(8):e1002628. doi: 10.1371/journal.pcbi.1002628 (PMC3415397; doi:10.1371/journal.pcbi.1002628)
Supplement: Text S4 — Complete source code for the PyDSTool package (version 0.88.120504). Includes API documentation and help files linking to web pages. This file is identical to the current public release on Sourceforge.net. (ZIP) [file pcbi.1002628.s004.zip › PyDSTool/html/toc-PyDSTool.PyCont.misc-module.html]

xml version="1.0" encoding="ascii"?


misc


# Module misc

---

## Classes

IterationError  
 Struct  

## Functions

CheckHopf  
 bialttoeig  
 bilinearform  

evec1\_standard

firstlyapunov  
 getFlowJac  
 getFlowMaps  
 getLeftEvecs  
 hess  
 hess3  
 ijtoind  
 indtoij  
 invwedge  
 isnotzero  
 iszero  
 jac  
 monotone  

negate

partition  
 testindij  
 tocoords  
 todict  
 trilinearform  
 unique  
 wedge  

## Variables

FLOQ\_TOL

Inf

NaN

PI

\_classes

\_functions

all\_curve\_types

all\_point\_types

c\_

conjugate

divide

isfinite

mod

multiply

r\_

sign

sqrt

subtract

---

[hide private]
